# Danhong Injection for the Treatment of Hypertensive Nephropathy: A Systematic Review and Meta-Analysis

**DOI:** 10.3389/fphar.2020.00909

**Published:** 2020-06-19

**Authors:** YiZhuo Li, Shihai Yan, Lichao Qian, Lihua Wu, Yawei Zheng, Zhuyuan Fang

**Affiliations:** ^1^Affiliated Hospital of Nanjing University of Chinese Medicine, Nanjing, China; ^2^Institute of Hypertension, Affiliated Hospital of Nanjing University of Chinese Medicine, Nanjing, China

**Keywords:** hypertensive nephropathy, Danhong injection, traditional Chinese medicine, meta-analysis, systematic review

## Abstract

**Objective:**

Danhong Injection (DHI) has been widely used to treat various diseases in China for many years. The objective of this systematic review was to evaluate the efficacy of DHI combined with antihypertensive drugs for treatment of hypertensive nephropathy.

**Methods:**

Seven databases were searched from inception to September 21st, 2019. Randomized controlled trials comparing DHI combined with antihypertensive drugs *versus* antihypertensive drugs alone were extracted. The primary outcome was microalbuminuria (mALB). Secondary outcomes included systolic blood pressure (SBP), diastolic blood pressure (DBP), and serum creatinine (SCr).

**Results:**

Fifteen studies were included in the meta-analysis, which indicated that DHI combined with antihypertensive drugs has advantages compared with antihypertensive drugs alone for reducing mALB [weighted mean difference (WMD) = −12.86, 95% confidence interval (CI) (−14.72, −11.0), *P* < 0.01], lowering SBP [WMD = −2.84, 95% CI (−4.56, −1.12), *P* = 0.001] and DBP [WMD = −2.38, 95% CI (−4.34, −0.43), P = 0.017], and decreasing SCr [WMD = −40.45, 95% CI (−55.69, −25.21), P < 0.01].

**Conclusion:**

The combination of DHI with antihypertensive drugs appears to be more effective than antihypertensive drugs alone for treatment of hypertensive nephropathy. A moderate duration (≤4 weeks) of DHI administration is reasonable, and longer treatment with DHI should be avoided, according to the results of subgroup analysis.

## Introduction

Hypertension is the leading preventable cause of premature death and disability worldwide (Mills et al., 2016). Further, high blood pressure (BP) is one of the most prevalent risk factors for cardiovascular and kidney diseases and has been identified as an important global health challenge (Kearney et al., 2005; Lawes et al., 2008). It is established that elevated BP expedites the progression of chronic kidney disease (CKD) (Rigo and Orias, 2019); hence the achievement of target BP is crucial for patients with CKD; however, numerous studies have demonstrated that the rate of BP control is quite low among patients with CKD (Plantinga et al., 2009), and the optimal target BP in patients with hypertensive nephropathy has been a point of debate for a long time. According to current guidelines, the target BP for patients with CKD is defined as <130/80 mmHg; however, there are no data from adequately powered randomized trials to support this (Hart and Bakris, 2010). The results of three trials, the Modification of Diet in Renal Disease (MDRD) study, Ramipril Efficacy in Nephropathy (REIN-2) trial and African-American Study of Kidney Disease and Hypertension (AASK) comparing two target BP levels (SBP < 140 mmHg for conventional control strategy and SBP < 130 mmHg for intensive control strategy) did not show a convincing benefit of a lower BP goal (Weir, 2014). Due to the difficulty in treating hypertensive nephropathy, hypertension has become the second leading cause of end-stage renal disease after diabetes (Williams et al., 2018). Given this situation, complementary therapies could be a good option for preventing the transition from hypertension to renal disease.

Microalbuminuria (mALB) is defined as urinary albumin excretion of >30–299 mg/d or 20–200 μg/min, and is a major marker of hypertensive nephrosclerosis, which reflects the loss of the glomerular filter selectivity (Bakris, 2004; Ibsen et al., 2005; Hart and Bakris, 2010; Seccia et al., 2017). Most trials have supported that lowering both BP and mALB can maximally delay the progression of nephropathy (Ruggenenti et al., 2004; Toto, 2005; Sarafidis et al., 2007). Traditional Chinese Medicine (TCM) is one of the most popular complementary therapies globally, and there is increasing evidence from animal and clinical studies to support the benefit of TCM in the treatment of hypertensive nephropathy (Ding et al., 2015; Wu et al., 2018; Yan et al., 2018).

DHI, manufactured by Buchang Pharmaceutical Company Limited (Shandong province, China), is a modern patented Chinese herbal medicine made from aqueous extracts of *Radix Salviae Miltiorrhizae* and *Flos Carthami tinctorii* with the raw material dose ratio of 3:1. Briefly, the production process involves: boiling *Radix Salviae Miltiorrhizae* and *Flos Carthami tinctorii* (ratio of 3:1) in distilled water three times (once for 2 h, then twice for 1.5 h), then concentrating the aqueous extracts to a specific concentration (Qingliang, 1987). Next, an ethanol precipitation process is used to remove impurities from the concentrated solution. Finally, the extract is dissolved in glucose solution and the pH value adjusted. All production process occur under strict aseptic conditions, and the quality control measures used for the process have been reported (Binjun et al., 2013; Yan et al., 2015; Zheng et al., 2015). DHI is formulated according to the theory of TCM and has been used, alone or in combination with other therapies, for treatment of a variety of diseases in China for many years (Sun et al., 2009; He et al., 2012; Zhang et al., 2016; Feng et al., 2019; You et al., 2019). Quality control and chemical analyses of DHI have also been reported, and the main effective components of DHI are identified as salvianolic acid B (0.51 ± 0.06 mg/ml), salvianolic acid A (0.31 ± 0.06 mg/ml), and hydroxysafflor yellow A (2.98 ± 0.41 μg/ml) (Zhang et al., 2016; Qi et al., 2017; Li et al., 2019; Xu et al., 2019). Previous studies have indicated that the dominant components of DHI have multiple effects, including vasodilation, decreasing vascular resistance and blood viscosity, recovering neurological function, reducing inflammatory responses, activating platelet inhibition, and improving BP (Feng et al., 2019). Meanwhile, DHI has been demonstrated to be an effective treatment for patients with hypertensive nephropathy; however, a systemic review of the effects of DHI for treatment of hypertensive nephropathy is lacking. In this meta-analysis, we systematically assessed the efficacy of DHI for the treatment of hypertensive nephropathy.

## Methods

This study was designed in accordance with the 2009 Preferred Reporting Items for Systematic Reviews and Meta-analysis (PRISMA) statement (Moher et al., 2009).

### Inclusion Criteria

Randomized controlled trials (RCTs), which compared DHI combined with antihypertensive drugs *versus* antihypertensive drugs alone, were eligible for inclusion, regardless of publication status, population characteristics, or language. Patients meeting the diagnostic criteria for hypertensive nephropathy, without other serious disease or complications, were included. There were no restrictions on patient sex, age, course of disease, or religion. Hypertension was defined as SBP ≥ 140 mmHg or diastolic blood pressure (DBP) ≥ 90 mmHg, and the diagnosis of hypertensive nephropathy was mainly based on elevated serum creatinine (SCr) and/or increased urinary albumin (James et al., 2014; Lisheng et al., 2019). Interventions in control groups included antihypertensive drugs and conventional therapies, such as benazepril, valsartan, irbesartan, amlodipine, nifedipine, compound *α*-ketoacid, *etc*.; there were no restrictions on dosage, type, frequency, or treatment course. Experimental group interventions were combinations of DHI and control group interventions. Placebo trials were also included.

### Exclusion Criteria

Studies were excluded if they were: (1) duplicated publications; (2) clinical trials where no relevant data could be extracted; (3) RCTs where participants had secondary hypertension, primary nephropathy, or diabetic nephropathy; (4) systematic reviews, important data reports, and case reports; or (5) clinical trials that failed to meet the inclusion criteria described above.

### Search Strategies

Databases, including Cochrane Library, PubMed, EMBASE, China National Knowledge Infrastructure (CNKI), Chinese Biomedical Database (CBM), VIP information resource integration service platform, and Wanfang Data Information Site, were searched from inception to September 21st, 2019. The search terms used were as follows: “hypertensive nephropathy”, “hypertensive renal injury”, “hypertensive kidney injury”, “danhong injection”, “danhong”, *etc* (see **Supplementary Table 1** for the search strategies).

### Study Selection and Data Extraction

The primary outcome was mALB levels, and secondary outcomes included SBP, DBP, and SCr. Study selection and data extraction were performed independently by two authors according to the search strategies. Preliminary screening was based on titles and abstracts of the results of all searches to exclude obviously unqualified studies. A full-text scan was then performed to assess whether the studies meet the inclusion criteria. Then, studies were cross-checked by two authors. Disagreements were resolved by discussion or consensus with a third author. Data (author’s name, publication year, participant information, intervention, duration, outcomes, and adverse events) were extracted into a spreadsheet.

### Assessment of Bias Risk of Included Studies

Two authors independently assessed the bias risk of included studies, according to the “Risk of bias” evaluation tool in the Cochrane Handbook for Systematic Reviews. The risk assessment contains seven items: (1) random sequence generation; (2) allocation concealment; (3) blinding of participants and personnel; (4) blinding of the outcome assessment; (5) incomplete outcome data; (6) selective reporting; (7) other bias. These items were evaluated as having a “high risk of bias,” “low risk of bias,” or “unclear risk of bias” according to the assessment criteria.

### Data Analysis

Statistical analysis was performed according to the guidelines of Cochrane Handbook for Systematic Review of Interventions (JPT et al., 2019), using Stata 14.0 software. Dichotomous variable were represented by relative risk (RR) and expressed by its 95% confidence interval (CI). For continuous variable, WMD or standardized mean difference (SMD), and 95% CI were used. Heterogeneity was evaluated statistically using the *I²* statistic and *χ²*. We adopted a fixed effect model, unless substantial heterogeneity was detected (*I²* > 50% or *P* < 0.05), then a random effects model was used. Subgroup analysis was performed to explore the potential causes of heterogeneity according to different interventions and baselines. *P* values < 0.05 were considered statistically significant. Potential publication bias was investigated by means of the Begger’s and Egger’s tests. Sensitivity analysis was performed in order to determine whether the conclusions would have differed if the eligibility were restricted to studies deemed at low risk of bias and the methods of data synthesis changed (such as changing the random-effects method to a fixed-effect model or altering the measures of treatment effects).

## Results

### Search Results

**Figure 1:**
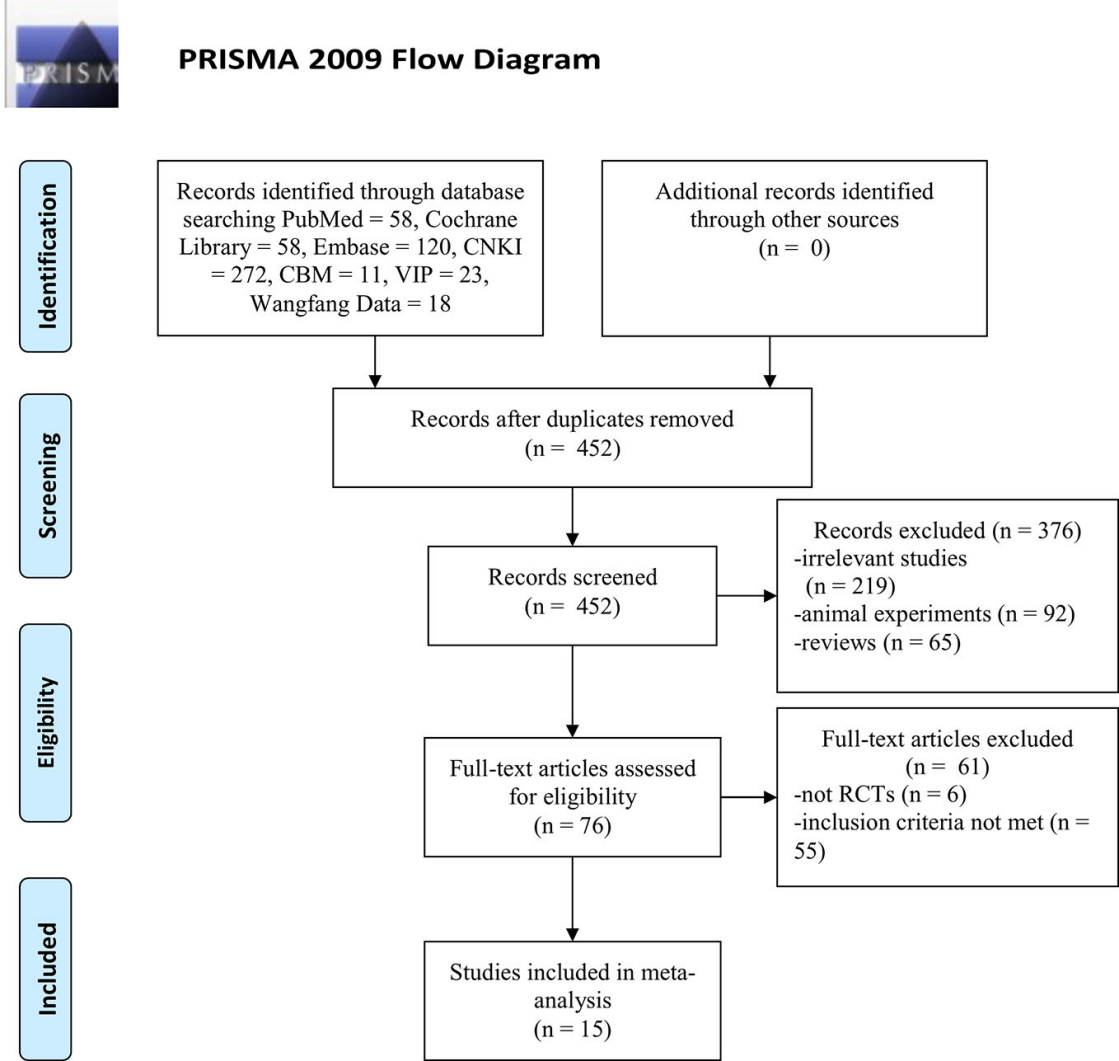
PRISMA 2009 Flow Diagram.

A total of 560 articles [the Cochrane Library (n = 58), PubMed (n = 58), Embase (n = 120), CBM (n = 11), CNKI (n = 272), Wanfang Data (n = 18), and VIP (23)] were retrieved, of which 108 studies were excluded because of duplicated publication. The titles and abstracts of the remaining 452 articles were screened, and 376 articles were excluded because of obvious ineligibility (219 irrelevant studies, 92 animal experiments, and 65 reviews). A further 61 articles were excluded after a full-text review. Among them, six articles were not RCTs (five cohort studies and one retrospective study), 55 articles did not meet the inclusion criteria (21 studies with no relevant data, 16 studies in which DHI combined with other drugs in experimental group, 15 studies that patients enrolled with primary nephropathy or diabetic nephropathy and three duplicated publication). Finally, fifteen articles remained and were included in the meta-analysis. The screening process is summarized as a PRISMA flow diagram (**Figure 1**).

### Study Characteristics

Fifteen randomized controlled trials (Changan and Meiyu, 2008; Guolong et al., 2010; Xiaoying, 2010; Cuixia, 2012; Wenhu, 2012; Jing et al., 2013; Shangping and Aihua, 2013; Liping, 2014; Xiaoping, 2014; Changjiang, 2015; Xuehua, 2015; Qingxin, 2016; Xuelian, 2016; Baobao, 2017; Youjun, 2017) involving 1,536 patients (785 and 751 in the experimental control groups) were included in this review. Characteristics of these studies were summarized in **Table 1**.

**Table 1:** Characteristics of the included studies.

Study	Sample size (T/C)	Sex M/F			Age(years)		Diagnosis standards	Intervention		Duration of treatment	Outcomes		Adverse events
		T		C	T	C		Treatment	Control					
Changan and Meiyu, 2008	52/40	41/11		32/8	71.0 ± 3.4	70.0 ± 3.2	Diagnosis and Treatment of Nephropathy (Ye Rengao)	DHI (40 ml ivgtt qd) + control (without Amino Acid Injection)	Antihypertension therapy (no details) + compound *α*-ketoacid (4 tablets tid) + Amino Acid Injection (250 ml ivgtt qd) + Low protein diet (0.6–0.8g/kg/d)	4 weeks	SCr, BUN	Not mentioned		
Guolong et al., 2010	102/102	70/32		75/27	68.7 ± 6.6	69.7 ± 7.8	GMAH (2007 ESH)	DHI (40 ml ivgtt qd) + control (without Amino Acid Injection)	Benazepril (10 mg/d) + Hydrochlorothiazide (12.5 mg/d) + Amino Acid Injection(250 ml ivgtt qd)	4 weeks	SCr, mALB, TC, LDL-C, Blood *β*_2_-MG, Urinary *β*_2_-MG, NAG, Ang II	Not mentioned		
Xiaoying, 2010	80/60	96/44 (no details)			77.0 ± 6.45	76.2 ± 6.75	CCSH (2010)	DHI (40 ml ivgtt qd) + control	Nifedipine (10 mg/d) + Hydrochlorothiazide (25 mg/d) + Telmisartan (40–80 mg/d)	4 weeks	SCr, Bun, Ccr, SBP, DBP	Not mentioned		
Cuixia, 2012	42/42	24/18		23/19	65.8 ± 14.7	63.4 ± 15.2	Internal Medicine (Lu Zaiying)	DHI (20 ml ivgtt qd) + control	Perindopril (4 mg/d) + Low protein, low salt and low fat diet (no details)	30 days	SCr, BUN, SBP, DBP, UAER	Not mentioned		
Wenhu, 2012	64/64	28/36		30/34	72.5 ± 12.8	73.0 ± 12.5	Essential hypertension with proteinuria	DHI (40 ml ivgtt qd) + control	Amlodipine (10 mg/d) + Hydrochlorothiazide (25 mg/d) + Valsartan (40 mg/d)	1 week	SCr, BUN, Ccr, SBP, DBP	Not mentioned		
Jing et al., 2013	31/34	39/33 (7 dropped, no details)			63.8 ± 9.4 (no details)		CGMH (1999)	DHI (40 ml ivgtt qd) + control	Amlodipine (5–10mg/d) + Hydrochlorothiazide (no details) + Metoprolol (no details)	4 weeks	mAlb, Urinary *β_2_*-MG, Urinary *α*_1_-MG, Urinary IgG,	T: 1 case of dizziness C: None		
Shangping and Aihua, 2013	30/30	40/20 (no details)			73.22 ± 10.56 (no details)		CGMH (2010)	DHI (40ml ivgtt qd) + control	Irbesartan (150-300mg/d)	6 weeks	mALB, Ccr, SBP, DBP, Urinary β_2_-MG	Not mentioned		
Liping, 2014	40/40	20/20	19/21		68.1 ± 7.9	67.8 ± 7.1	CGMH (2010)	DHI (40ml ivgtt qd) + control	Irbesartan (150-300mg/d)	6 weeks	mALB, Ccr, SBP, DBP, Urinary β_2_-MG	Not mentioned		
Xiaoping, 2014	32/32	42/22 (no details)			58.3 ± 11.7 (no details)		CGMH (1999)	DHI (40 ml ivgtt qd) + control	Amlodipine (5–10 mg/d)	4 weeks	mAlb, Urinary *β*2-MG, Urinary *α*1-MG, Urinary IgG,	Not mentioned		
Changjiang, 2015	49/49	63/35 (no details)			71.2 ± 10.9 (no details)		CGMH (2010)	DHI (40 ml ivgtt qd) + control	Nifedipine (10 mg/d) + Hydrochlorothiazide (20 mg/d) + losartan (40 mg/d)	4 weeks	SCr, Bun, Ccr, SBP, DBP	Not mentioned		
Xuehua, 2015	34/34	45/23 (no details)			65.4 ± 2.3 (no details)		CGMH (2010)	DHI (40 ml ivgtt qd) + control	Irbesartan (150 mg/d)	30 days	mALB, Ccr, SBP, DBP, Urinary *β*_2_-MG	Not mentioned		
Xuelian, 2016	40/40	24/16	22/18		62.6 ± 9.1	63.4 ± 9.6	CGMH (2010)	DHI (40 ml ivgtt qd) + control	Benazepril (no details) + Quitting cigarettes and alcohol + Low protein, low salt and low fat diet (no details)	4 weeks	CE, SCr, Bun, 24 h UTP, mALB, Hemorheology	Not mentioned		
Qingxin, 2016	100/100	70/30	75/25		68.7 ± 6.5	69.7 ± 7.4	CGMH (2010)	DHI (40 ml ivgtt qd) + control	Benazepril (10 mg/d)	4 weeks	mALB, Blood *β*2-MG, Urinary *β*_2_-MG, NAG, Ang II	Not mentioned		
Youjun, 2017	36/36	21/15	20/16		60.8 ± 7.5	60.4 ± 7.3	CGMH (2010)	DHI (40 ml ivgtt qd) + control	Valsartan (80 g/d)	8 weeks	CE, mALB, 24h UTP, Fibrinogen, Plasma viscosity	Not mentioned		
Baobao, 2017	53/48	28/25	25/23		49.6 ± 18.7	48.8 ± 18.9	Nephrology (Wang Haiyan)	DHI (40 ml ivgtt qd) + control	Antihypertension therapy (no details) + Trimetazidine (20 mg tid) + Low protein, low salt and low fat diet (no details)	2 weeks	CE, mALB, SCr, BUN, Ccr, 24 h UTP, GFR, CysC, SBP, DBP, ET-1, NO	Not mentioned		

T, treatment group; C, control group; BUN, blood urea nitrogen; GMAH (2007 ESH), Guidelines for the management of arterial hypertension (2007 European society of cardiology); TC, total cholesterol; LDL-C, low density lipoprotein cholesterol; β2-MG, beta-2-microglobulin; NAG, N-acetyl-beta-D-glucosaminidase; Ang II, angiotensin II; CCSH (2010), Consensus of Chinese senior hypertension (2010); Ccr, creatinine clearance rate; UAER, urinary protein excretion rate; CGMH (1999), 1999 Chinese guidelines for the management of hypertension; α1-MG, alpha-1- macroglobulin; IgG, immune globulin G; CGMH (2010), 2010 Chinese guidelines for the management of hypertension; CE, clinical efficacy; 24 h UTP, 24-hours urinary total protein; GFR, glomerular filtration rate; CysC, cystatin C; ET-1, endothelin-1; NO, nitric oxide.

### Risk of Bias in Included Studies

**Figure 2:**
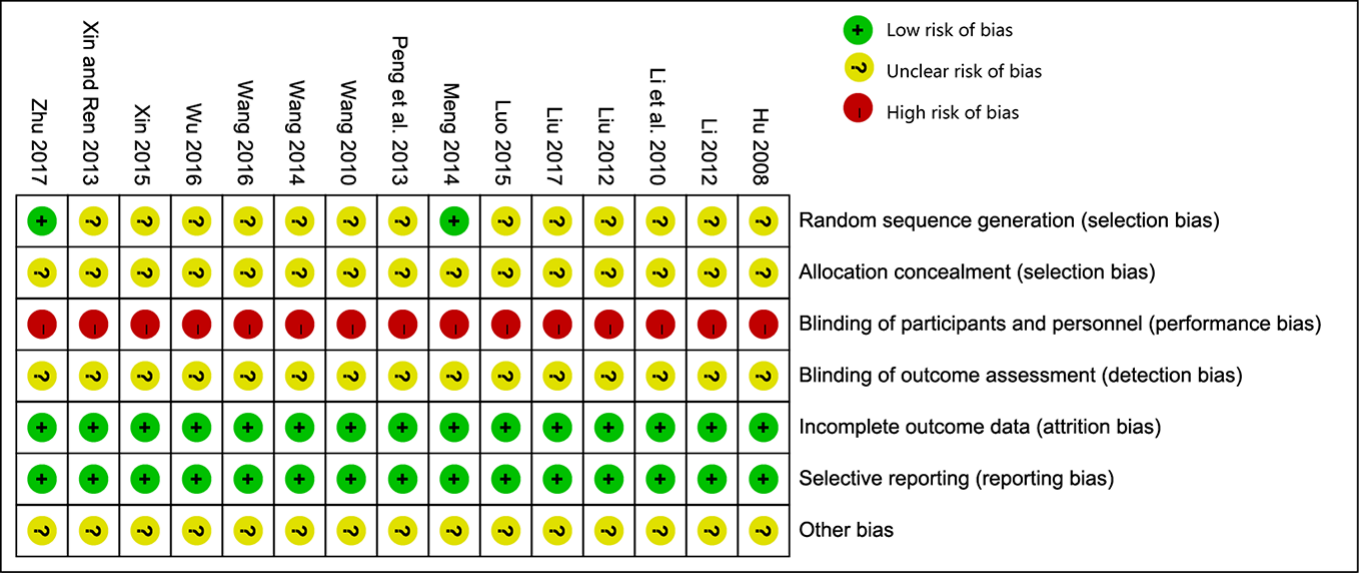
Risk of bias summary.

All of the 15 studies mentioned randomized allocation, while only two studies (Xiaoping, 2014; Youjun, 2017) explained the specific allocation methods. There was no information about allocation concealment, blinding, or evaluator blinding in any of these studies. Quality assessment is presented in **Figure 2**.

### Outcomes Measures

#### Microalbuminuria

**Figure 3:**
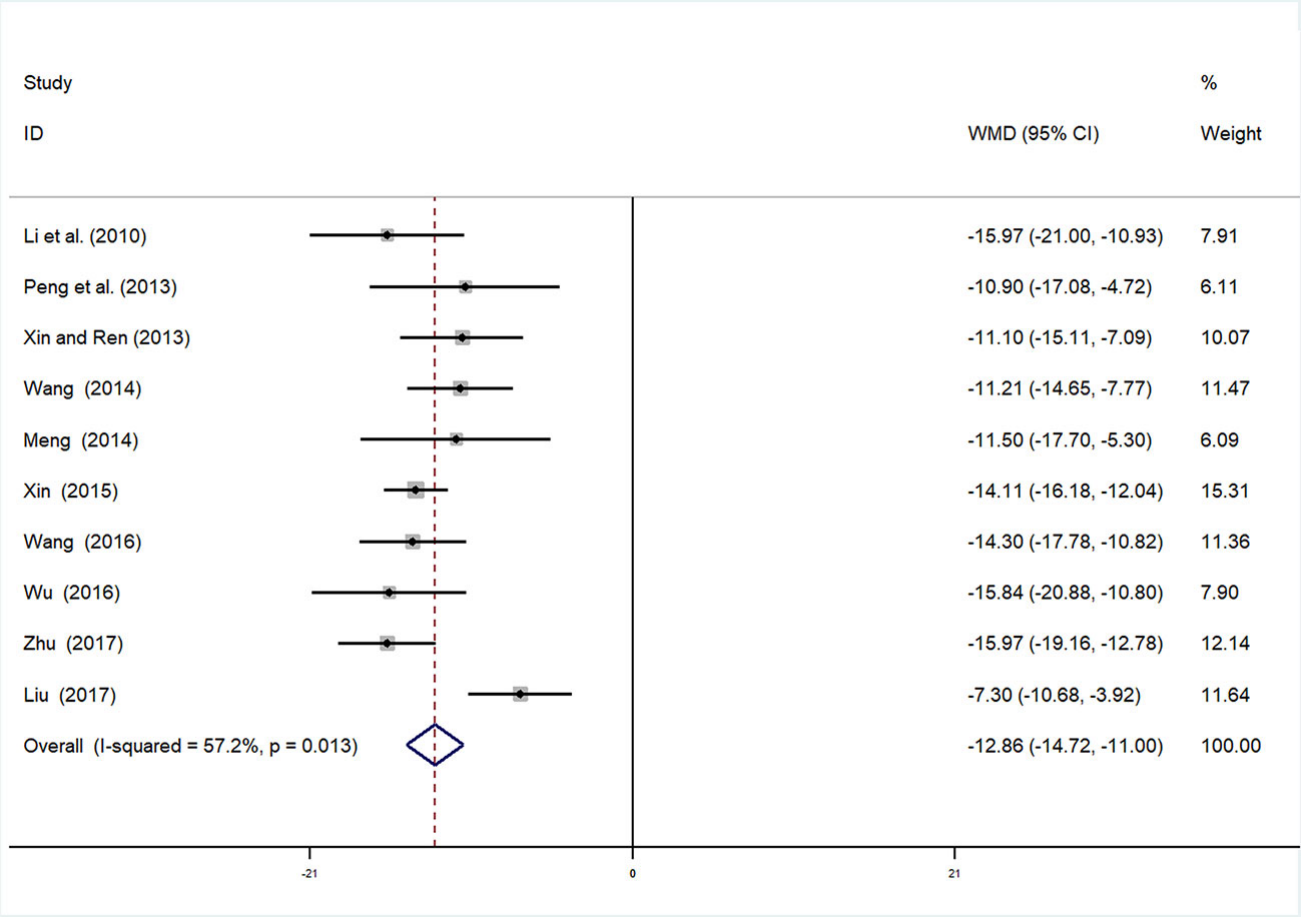
Forrest plot of mALB.

**Figure 4:**
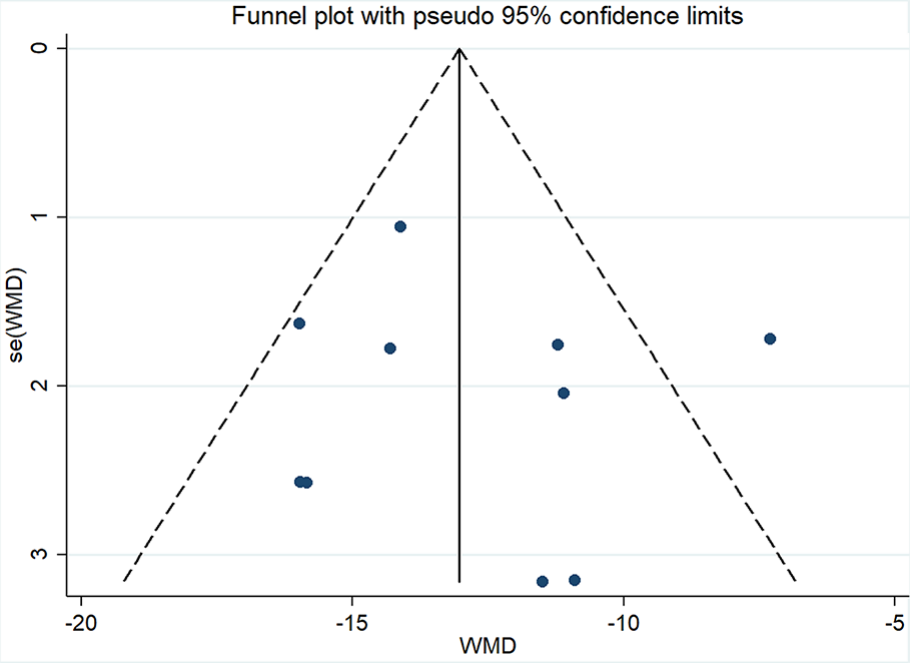
Funnel plot for publication bias of mALB.

Ten studies (Guolong et al., 2010; Jing et al., 2013; Shangping and Aihua, 2013; Liping, 2014; Xiaoping, 2014; Xuehua, 2015; Qingxin, 2016; Xuelian, 2016; Baobao, 2017; Youjun, 2017) involving 994 participants reported mALB. After testing for heterogeneity (*I^2 =^* 57.2%, *P* = 0.013, **Figure 3**), a random-effects model was adopted. A funnel plot analysis of the 10 trials suggested possible publication bias and inclusion of low quality studies, as asymmetry was shown (**Figure 4****)**. Begger’s test (*P* = 0.858) and Egger’s test (*P* = 0.690) (**Supplementary Figures 1** and **2**) were conducted and indicated that there was no publication bias (*P* values > 0.05). Meta-analysis indicated that the experimental group performed better than the control group in reducing mALB [WMD = −12.86, 95% CI (−14.72, −11.0), *P* < 0.01] (**Figure 3**, **Supplementary Figure 3****)**.

##### Sensitivity Analysis of mALB

A sensitivity analysis for mALB was performed **(****Supplementary Figure 4****)**. We serially excluded one trial each time and re-performing meta-analysis of the remaining trials to determine whether the outcomes had significantly changed. Sensitivity analysis revealed that the outcomes of mALB were very similar, indicating that the results were relatively stable.

##### Subgroup Analysis of mALB

**Figure 5:**
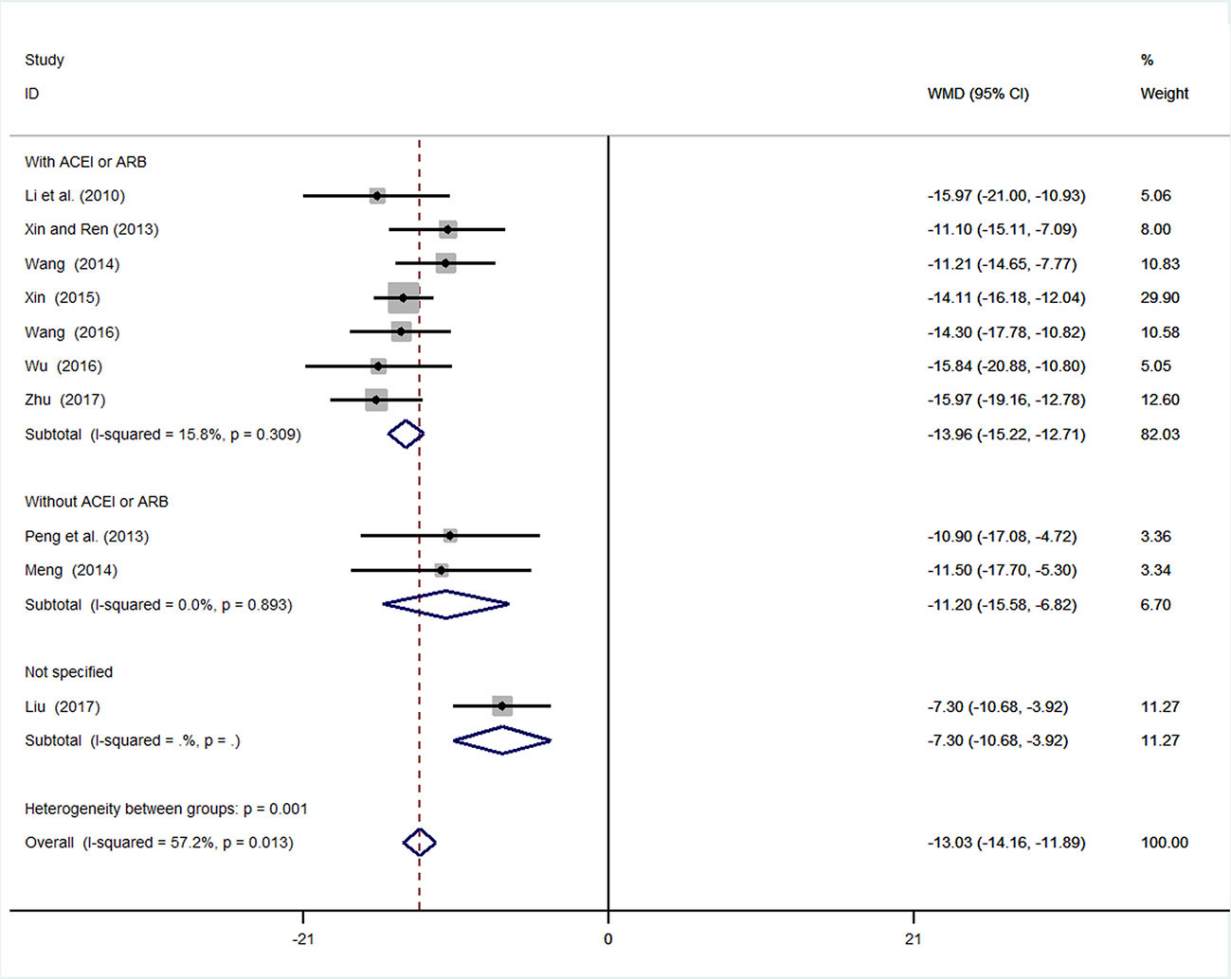
Subgroup analysis of mALB.

We conducted subgroup analysis (employing a fixed-effects model) of mALB because of the high heterogeneity. Many previous trials have revealed that inhibition of the renin–angiotensin system (RAS) by angiotensin converting enzyme inhibitors (ACEI) or angiotensin receptor blockade (ARB) is superior to conventional therapy for preserving renal function (Jafar et al., 2001; Rahman et al., 2005). In these ten studies, two used other antihypertension drugs (amlodipine), and one study did not mention the specific types of antihypertensive drugs used. Subgroup analysis was performed based on whether ACEI or ARB was used. In the subgroup with ACEI or ARB, the result of meta-analysis was statistically significant with low heterogeneity [WMD = −13.96, 95% CI (−15.22, −12.71), *P* < 0.01; *I^2^* = 15.8%]. In the subgroup without ACEI or ARB, the result of meta-analysis remained statistically significant with low heterogeneity [WMD = −11.20, 95% CI (−15.58, −6.82), *P* < 0.01; *I^2^* = 0%] (**Figure 5**, **Supplementary Figure 5****)**.

#### Systolic Blood Pressure

**Figure 6:**
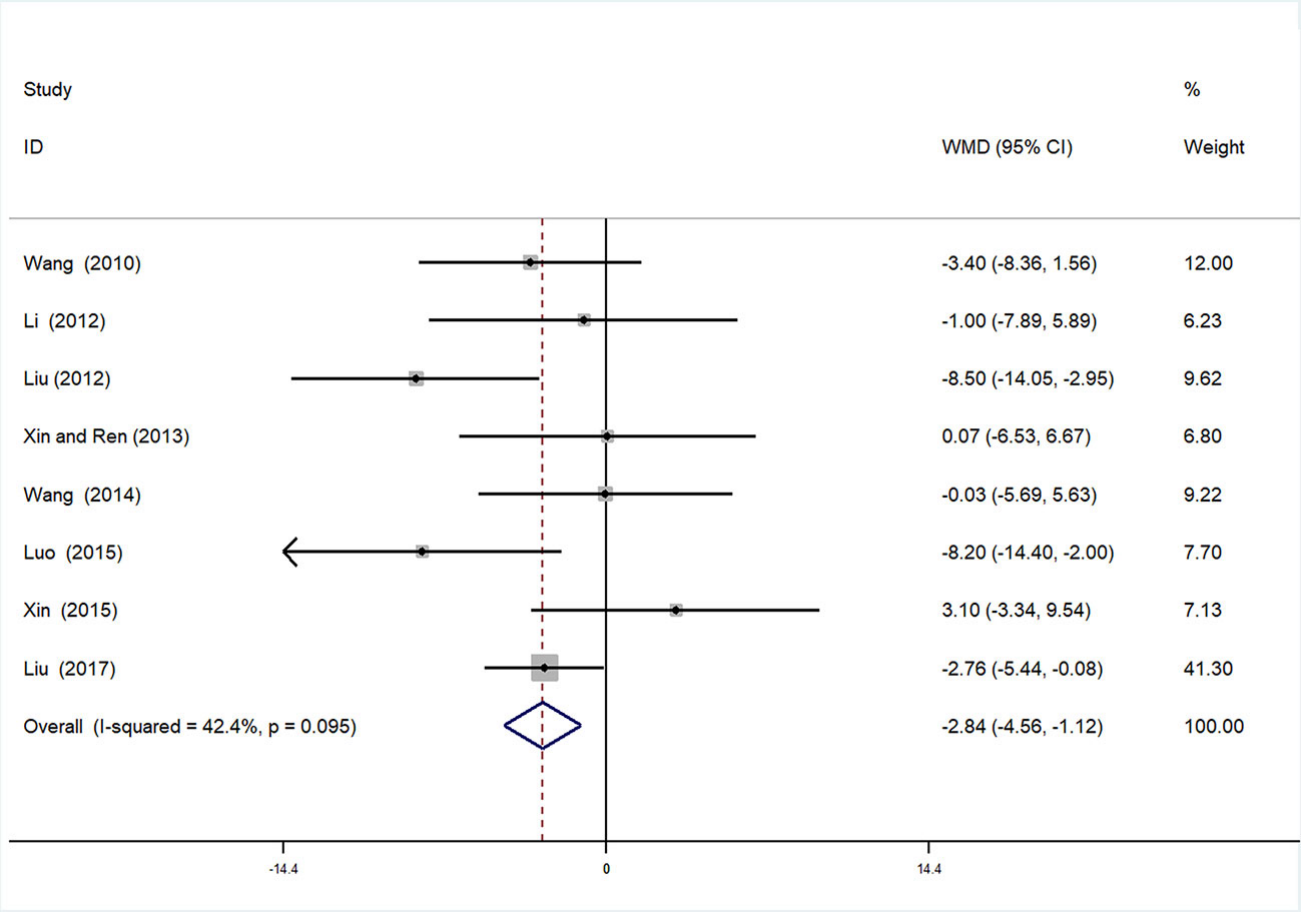
Forrest plot of SB.

Eight studies (Xiaoying, 2010; Cuixia, 2012; Wenhu, 2012; Shangping and Aihua, 2013; Liping, 2014; Changjiang, 2015; Xuehua, 2015; Baobao, 2017) involving 759 participants contributed to this analysis. After testing for heterogeneity (*I^2^* = 42.4%, *P* = 0.095, **Figure 6**), we employed a fixed-effects model. Begger’s test (*P* = 0.536) and Egger’s test (*P* = 0.835) were conducted to assess publication bias for SBP (**Supplementary Figures 6** and **7**). Sensitivity analysis of SBP was also performed, and by serial exclusion of one trial each time and re-performing meta-analysis of the remaining trials, we observed that the outcomes of SBP were very similar, indicating that the result was relatively stable **(****Supplementary Figure 8****)**. Meta-analysis revealed a statistically significant difference between the experimental and control groups [WMD = −2.84, 95% CI (−4.56, −1.12), *P* = 0.001] (**Figure 6**, **Supplementary Figure 9****)**.

##### Subgroup Analysis of SBP

**Figure 7:**
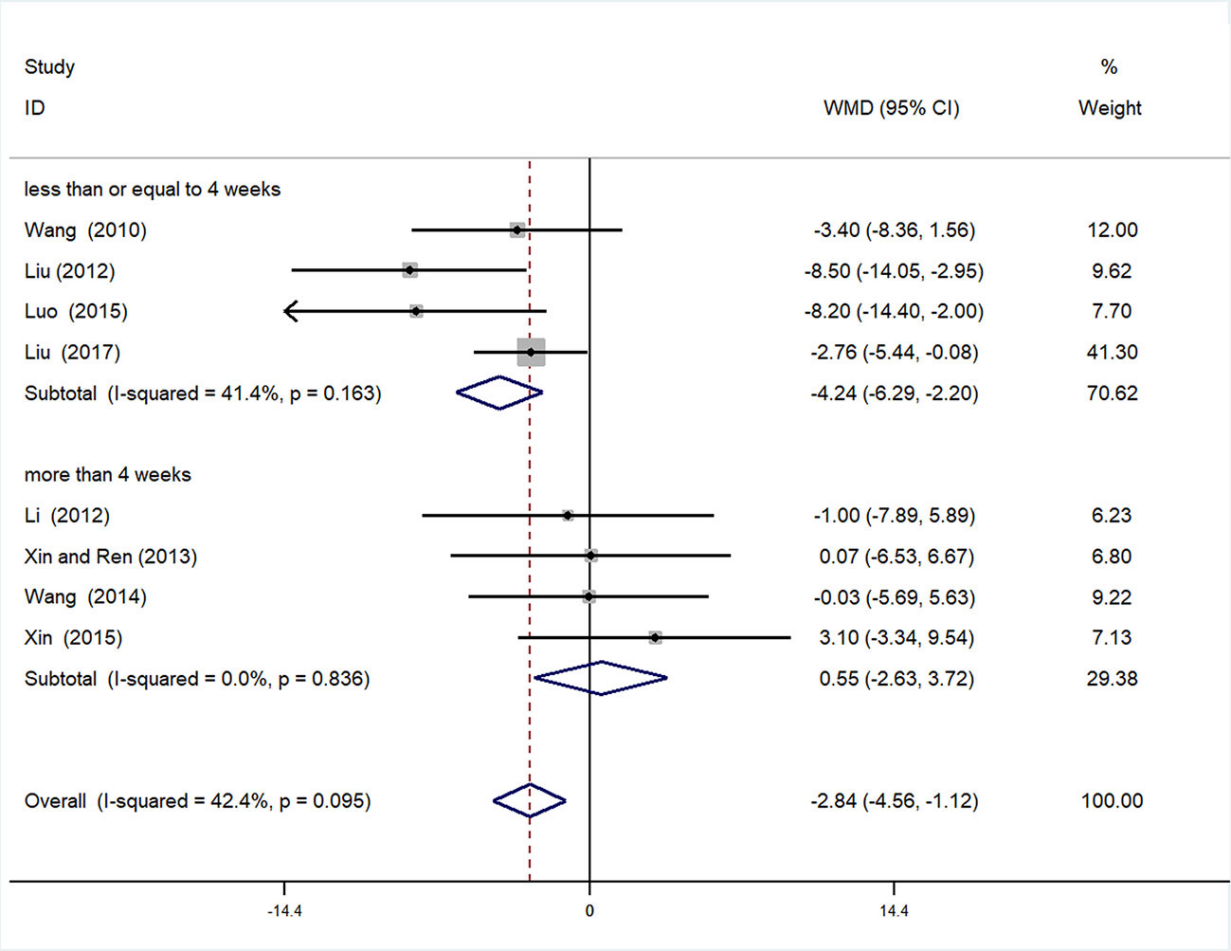
Subgroup analysis of SBP.

Subgroup analysis of SBP was conducted based on different duration of treatment (>4 or ≤4 weeks). In the >4 weeks treatment duration subgroup, meta-analysis indicated a nonsignificant trend for reduction in SBP between experimental and control groups [WMD = 0.55, 95% CI (−2.63, 3.72), *P* = 0.735]; however, in the subgroup with treatment duration ≤ 4 weeks, there was a significant trend for reduction between the two groups [WMD = −4.42, 95% CI (−6.29, −2.20) *P* < 0.01] (**Figure 7**, **Supplementary Figure 10****)**.

#### Diastolic Blood Pressure

**Figure 8:**
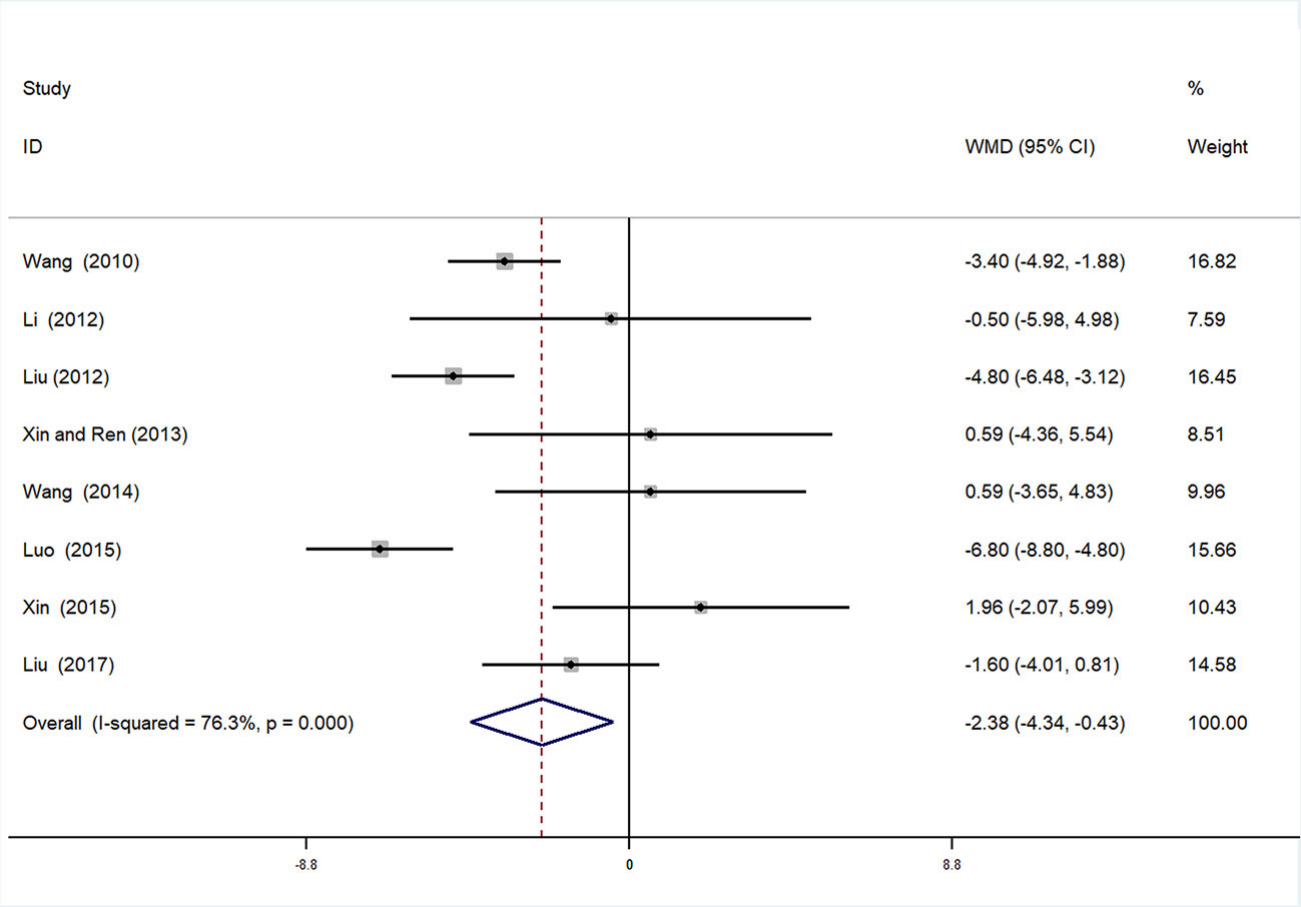
Forrest plot of DBP.

A total of eight studies (Xiaoying, 2010; Cuixia, 2012; Wenhu, 2012; Shangping and Aihua, 2013; Liping, 2014; Changjiang, 2015; Xuehua, 2015; Baobao, 2017) were incorporated in the analysis of DBP. After testing for heterogeneity (*I^2^* = 76.3%, *P* < 0.01, **Figure 8**), a random-effects model was employed. We conducted Begger’s (*P* = 0.536) and Egger’s (*P* = 0.053) tests to assess publication bias **(****Supplementary Figures 11** and **12**). Given the high heterogeneity, sensitivity analysis of DBP data was performed. By seriatim excluding one trial each time and re-performing meta-analysis of remaining trials, we observed that the outcomes of DBP were very similar, which means that the result was relatively stable **(****Supplementary Figure 13****)**. Meta-analysis revealed that the experimental group performed better than control group in reducing DBP [WMD = −2.38, 95% CI (−4.34, −0.43), P = 0.017] (**Figure 8**, **Supplementary Figure 14****)**.

##### Subgroup Analysis of DBP

**Figure 9:**
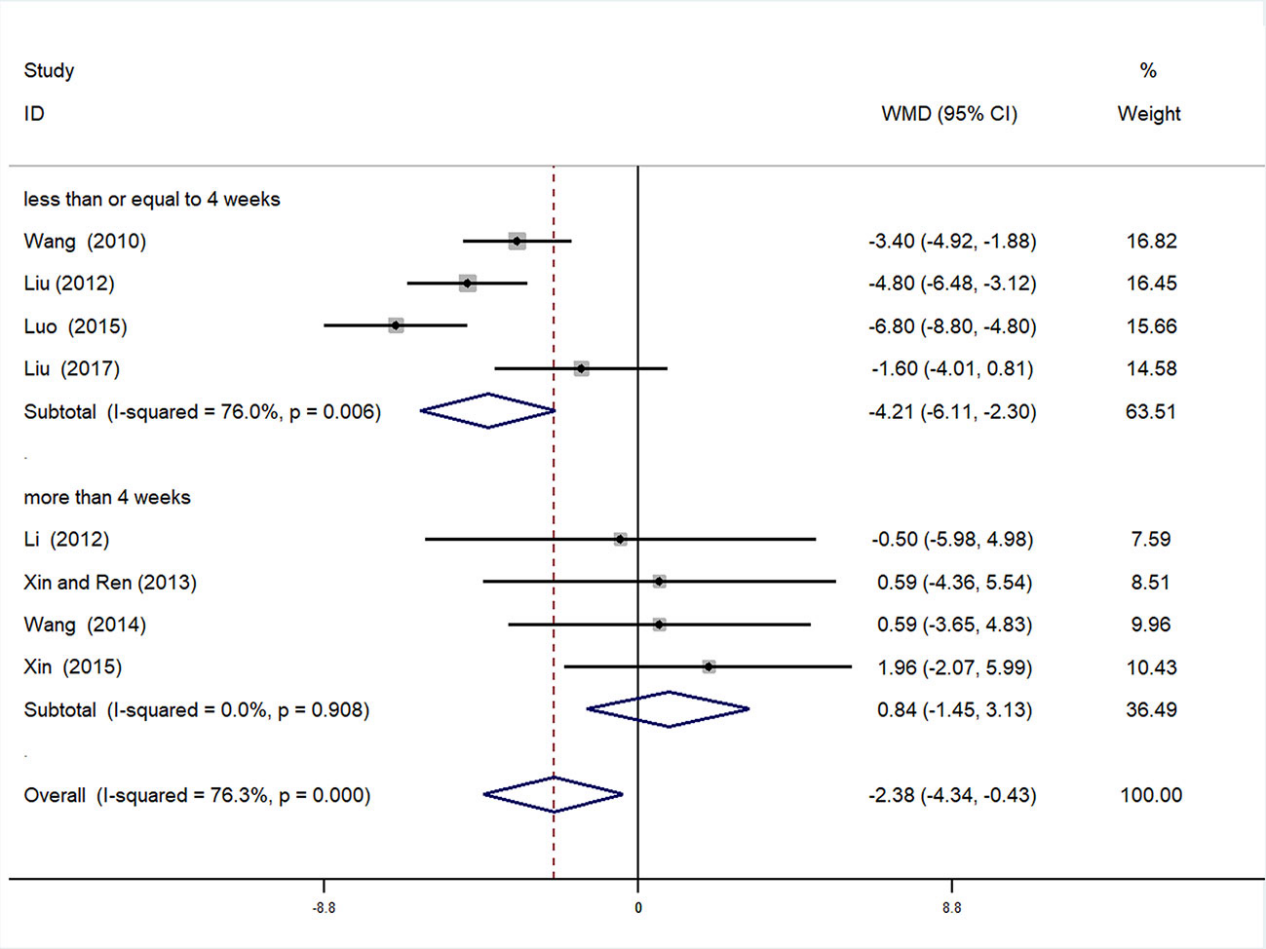
Subgroup analysis of DBP.

We also conducted subgroup analysis of DBP, according to treatment duration (>4 weeks or ≤4 weeks). Meta-analysis of the >4 weeks treatment duration subgroup indicated a nonsignificant trend toward reduction in DBP between experimental and control groups [WMD = 0.84, 95% CI (−1.45, 3.13), *P* = 0.471], while there was a significant decrease in DBP of the experimental group compared with the control group in the ≤4 weeks subgroup [WMD = −4.21, 95% CI (−6.11, −2.30), *P* < 0.01] (**Figure 9**, **Supplementary Figure 15****)**.

#### Serum Creatinine

**Figure 10:**
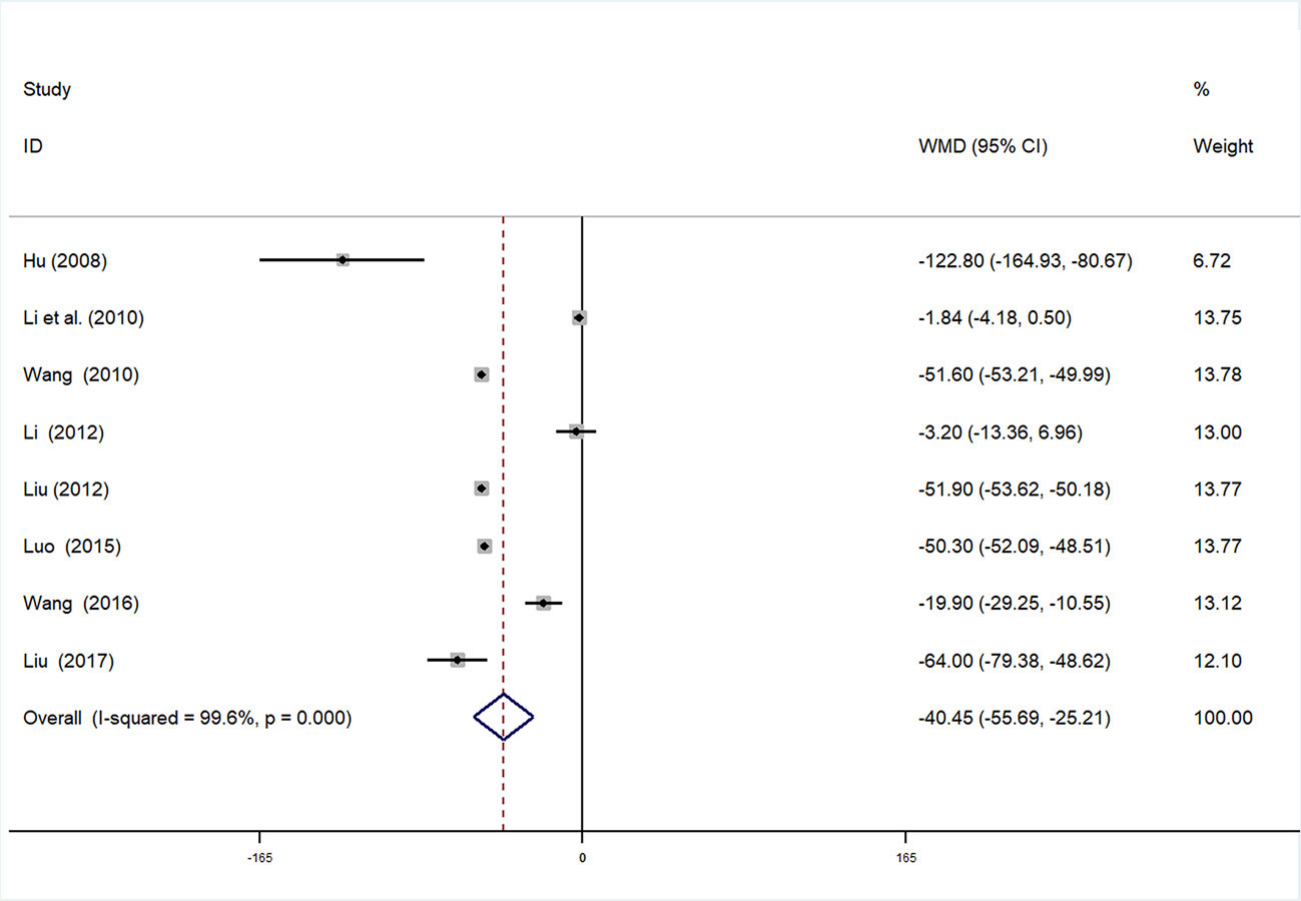
Forrest plot of Scr.

SCr data were available in eight studies (Changan and Meiyu, 2008; Guolong et al., 2010; Xiaoying, 2010; Cuixia, 2012; Wenhu, 2012; Liping, 2014; Changjiang, 2015; Xuelian, 2016; Baobao, 2017) involving 927 participants. After testing for heterogeneity (*I^2^* = 99.6%, *P* < 0.01, **Figure 10**), we adopted a random-effects model. Begger’s (*P* = 0.266) and Egger’s (*P* = 0.625) tests were conducted to evaluate publication bias **(****Supplementary Figures 16** and **17**). As high heterogeneity was detected, sensitivity analysis of SCr was performed. By serial exclusion of one trial each time and re-performing meta-analysis of the remaining trials, we observed that the outcomes of SCr were very similar, indicating that the result was relatively stable **(****Supplementary Figure 18****)**. Meta-analysis revealed that the experimental group performed better than control group in reducing SCr [WMD = −40.45, 95% CI (−55.69, −25.21), P < 0.01] (**Figure 10**, **Supplementary Figure 19****)**.

## Discussion

This meta-analysis was based on 15 RCTs, with a total of 1,536 participants with hypertensive nephropathy. The main findings can be summarized as follows: (1) mALB: There was a significant advantage of DHI combined with antihypertensive drugs compared with antihypertensive drugs alone for patients with hypertensive nephropathy in terms of reducing mALB. Subgroup analysis of mALB was conducted according to different types of antihypertensive drugs due to high heterogeneity among included studies. The benefit of DHI for patients with hypertensive nephropathy was observed in all subgroups with low heterogeneity. Hence, we speculate that the differences in antihypertensive drugs were the potential source of heterogeneity. The inhibition of RAS has been proved to be highly effective in slowing the progression of renal disease in previous studies (Jafar et al., 2001; Rahman et al., 2005). A trend (but not significant) in favor of DHI combined with ACEI or ARB over combination of DHI with other antihypertensive drugs was observed in our subgroup analysis. (2) SBP and DBP: SBP and DBP levels were significantly lower in patients treated with DHI compared with those of the control group. It was noted that in the subgroup analysis based on duration of treatment, no significant differences in SBP and DBP were observed between the experimental and control groups treated more than 4 weeks. We considered that 4 weeks may be a key point as the majority of patients achieved the BP goal on this stage. Therefore, a longer term treatment with DHI might be unreasonable. However, due to the poor methodological quality of these included RCTs, the placebo effect should be considered, and the reliability of this conclusion should be tested by prospective studies. (3) SCr: This meta-analysis also showed a statistically significant benefit of DHI combined with antihypertensive drugs over antihypertensive drugs alone in terms of the level of SCr; however, these findings should be interpreted with great caution, given the detection of high heterogeneity.

Hypertensive nephropathy is a common complication of hypertension, and current treatments do not greatly improve patient prognosis. Hence, identification of other effective therapies for patients with hypertensive nephropathy is important. DHI, which comprises aqueous extracts of *Radix Salviae Miltiorrhizae* and *Flos Carthami tinctorii*, was initially designed to treat cardiovascular and cerebrovascular diseases; however, increasing evidence indicates the benefit of DHI on nephropathy and hypertension (Liu et al., 2015; Feng et al., 2019), leading many clinicians to attempt use of DHI to treat hypertensive nephropathy.

The classical pathogenesis of hypertensive nephropathy is associated with arteriole vasoconstriction and hyalinosis caused by damage in the capillary tuft due to high BP, with the inappropriate activation of RAS (Seccia et al., 2017). Arteriole vasoconstriction results in capillary hypertension, which in turn gives rise to glomerular hyperfiltration and hypertrophy (Kriz and Lemley, 2015). Hyperfiltration increases fluid shear stress and damages podocyte, which leads to blunting of the selectivity of the glomerular filtration barrier (Seccia et al., 2017). A previous study suggested that DHI exhibits vasodilatory effect by inhibiting voltage-dependent Ca^2+^ release and inositol-3-phosphate (IP3) receptor-mediated Ca2^+^ influx (Zhi et al., 2012). This effect of DHI could alleviate glomerular hyperfiltration and decrease mALB. It is well known that kidney is one of the most vascularized organs in the body, and the integrity of the endothelial layer is important for many aspects of vascular function. In patients with hypertensive nephropathy, the endothelium has a pivotal role, not only in cardiovascular morbidity and mortality but also with regard to disease progression (Mennuni et al., 2014). In an *in vitro* study, DHI promoted the migration of endothelial progenitor and thereby vascular endothelium injury repair by upregulating the expression of Akt, eNOS, and MMP-9 (Hu et al., 2019).

In the TCM theory, hypertensive nephropathy is classified as “edema” or “dysuria and vomiting”. And “blood stasis” is the common TCM syndrome (also called “zheng” or “pattern”) of hypertensive kidney damage (Xiong et al., 2013; Cong et al., 2019). Therefore, “promoting blood circulation to remove blood stasis” is the principle for treatment of hypertensive nephropathy (Xiong et al., 2013). DHI is formulated based on the TCM theory of “activating and promoting blood circulation to dissipate blood stasis and dredge collaterals” (Owoicho Orgah et al., 2018). Although the indications of DHI focus on cardiovascular and cerebrovascular diseases, “blood stasis” is the common TCM “zheng” of hypertensive nephropathy and cardiovascular diseases. According to the TCM theory “treating different diseases (similar ‘zheng’) with the same method”, DHI may have a positive role for the treatment of hypertensive nephropathy, as supported by the results of this meta-analysis.

### Limitations

Several limitations should be taken into consideration. First, the methodological quality of the included studies was generally low. All of these studies claimed to be randomized, but only two reports mentioned sequence generation. There was no information about allocation concealment for any of the 15 studies. Hence, effective randomization is doubtful, which could lead to potential selection bias. Second, blinding is an essential method for preventing research outcomes from being influenced by either the placebo effect or the observer bias. However, none of the studies mentioned blinding of participants, personnel, or outcome assessment. Third, all of the included trials were conducted in China, which could lead to potential location bias. Finally, the diagnosis criteria of hypertensive nephropathy are not currently standardized across the world. Hence, the different diagnosis criteria adopted in the included studies may have influenced their reliability. Despite these limitations, this investigation is the first systematic assessment of the efficacy of DHI on hypertensive nephropathy, which could be helpful for clinicians.

## Conclusions

Evidence from this meta-analysis suggests that DHI combined with antihypertensive drugs may be more effective than the use of antihypertensive drugs alone for treating hypertensive nephropathy. DHI combined with antihypertensive drugs is associated with a reduction in albuminuria and a decrease in BP in patients with hypertensive nephropathy. A moderate duration (≤4 weeks) of DHI treatment is reasonable, while over long term exposure should be avoided, according our subgroup analysis. Nevertheless, the methodological quality of the RCTs included is relatively low; therefore, our conclusions should be interpreted with caution. Prospective, rigorously designed, large-scale, and multicenter clinical trials are still needed to assess the efficacy of DHI for treatment of hypertensive nephropathy and to find an optimal duration for treatment with DHI in the future.

## Author Contributions

YL, SY and ZF initiated this study and participated in its design. YL, LQ and YZ performed study selection, data extraction, and data analysis. YL and LW drafted the manuscript. SY and ZF supervised all aspects of the study. All authors contributed to the article and approved the submitted version.

## Funding

This work was supported by the National Science Foundation of China (81873258), a Project Funded by the Priority Academic Program Development of Jiangsu Higher Education Institutions (PAPD), The Open Projects of the Discipline of Chinese Medicine of Nanjing University of Chinese Medicine Supported by the Subject of Academic priority discipline of Jiangsu Higher Education Institutions (NO.ZYX03KF073) and Jiangsu Province “333 Talents Project” (2018-III-2195).

## Conflict of Interest

The authors declare that the research was conducted in the absence of any commercial or financial relationships that could be construed as a potential conflict of interest.
