# Supplementary material for: Danhong Injection for the Treatment of Hypertensive Nephropathy: A Systematic Review and Meta-Analysis
Source: Front Pharmacol. 2020 Jun 19;11:909. doi: 10.3389/fphar.2020.00909 (PMC7316888; doi:10.3389/fphar.2020.00909)
Supplement: Supplementary file 1 [file DataSheet_1.pdf]

Supplementary Materials for
"Danhong Injection for the Treatment of Hypertensive Nephropathy: A Systematic Review and Meta-Analysis"

YiZhuo Li[1], Shihai Yan[1], Lichao Qian[1], Lihua Wu[1], Yawei Zheng[1], and Zhuyuan Fang[2*]

1 *Affiliated Hospital of Nanjing University of Chinese Medicine, Nanjing, Jiangsu 210029, China*

2 *Institute of Hypertension, Affiliated Hospital of Nanjing University of Chinese Medicine, Nanjing, Jiangsu 210029, China*

*Corresponding author, Zhuyuan Fang. *E-mail address*: jsszyyfzy@163.com .

Table. 1. Search strategies

Databases	Search items	Result
Cochrane Library	#1 (hypertension):ti,ab,kw OR (hypertensive nephropathy) OR (hypertension nephropathy) OR (hypertensive renal injury) OR (hypertensive kidney injury) (Word variations have been searched)<br>#2 (hypertensive renal damage) OR (hypertensive kidney damage)<br>#3 #1 OR #2<br>#4 (danhong) OR (danhong injection) OR (Salvia) OR (safflower)<br>#5 #3 AND #4	58
Pubmed	((((((((danhong[Title/Abstract]) OR danhong injection[Title/Abstract]) OR Salvia[Title/Abstract]))) OR safflower[Title/Abstract])) AND ((hypertension[Mesh) OR ((((((hypertensive nephropathy[Title/Abstract]) OR hypertension nephropathy[Title/Abstract]) OR hypertensive renal injury[Title/Abstract]) OR hypertensive kidney injury[Title/Abstract]) OR hypertensive renal damage[Title/Abstract]) OR hypertensive kidney damage[Title/Abstract])))	58
EMBASE	#1 danhong:ab,ti OR 'danhong injection':ab,ti OR salvia:ab,ti OR safflower:ab,ti<br>#2 hypertension:ab,ti OR 'hypertensive nephropathy':ab,ti OR 'hypertension nephropathy':ab,ti OR 'hypertensive renal injury':ab,ti OR 'hypertensive kidney injury':ab,ti OR 'hypertensive renal damage':ab,ti OR 'hypertensive kidney damage':ab,ti<br>#3 #1 and #2	120
CNKI	FT=('高血压肾损害'+'高血压肾病'+'高血压肾功能不全'+'高血压性肾损害')*('丹红注射液'+'丹红')	272
CBM	#1 ((("高血压肾病"[常用字段]) OR "高血压肾损害"[常用字段]) OR "高血压肾功能不全"[常用字段]) OR "高血压性肾病"[常用字段]<br>#2("丹红注射液"[常用字段]) OR "丹红"[常用字段]<br>#1 AND #2 (("丹红注射液"[常用字段]) OR "丹红"[常用字段]) AND (((("高血压肾病"[常用字段]) OR "高血压肾损害"[常用字段]) OR "高血压肾功能不全"[常用字段]) OR "高血压性肾病"[常用字段])	11
VIP	(U=高血压肾损害 OR 高血压肾病 OR 高血压肾功能不全 OR 高血压性肾病）AND (U=丹红注射液 OR 丹红)	23
Wanfang Data	全部: (("高血压肾损害" + "高血压肾病"+"高血压肾功能不全"+"高血压性肾病") * ("丹红注射液" + "丹红"))	18

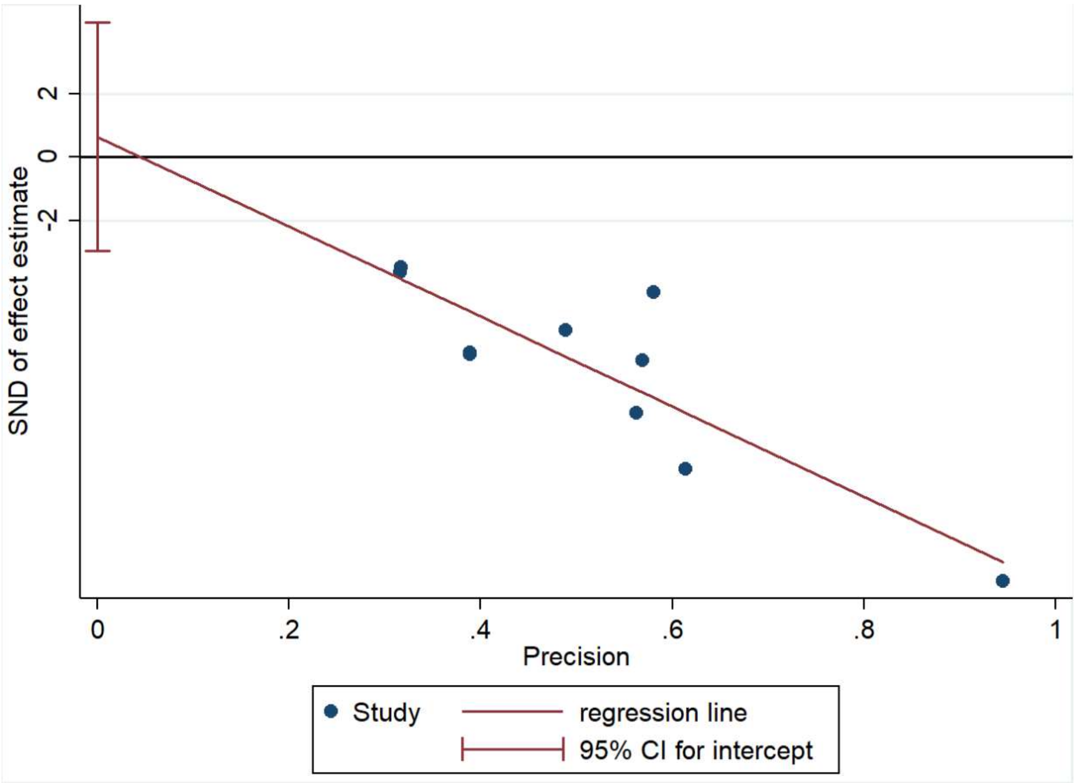

Supplementary Fig. 1. Egger's test for mALB

```
Regress standard normal deviate of intervention
effect estimate against its standard error

Number of studies =  10                          Root MSE      =   1.604
------------------------------------------------------------------------------
     Std_Eff |      Coef.   Std. Err.       t    P>|t|     [95% Conf. Interval]
-------------+----------------------------------------------------------------
       slope |  -14.14515   2.854156    -4.96    0.001    -20.72684   -7.563451
        bias |   .6457203   1.560583     0.41    0.690    -2.952992    4.244432
------------------------------------------------------------------------------

Test of H0: no small-study effects          P = 0.690

. metabias _ES _seES, begg

Note: data input format theta se_theta assumed.

Begg's test for small-study effects:
Rank correlation between standardized intervention effect and its standard error

  adj. Kendall's Score (P-Q) =        3
          Std. Dev. of Score =    11.18
           Number of Studies =       10
                           z =     0.27
                    Pr > |z| =    0.788
                           z =     0.18 (continuity corrected)
                    Pr > |z| =    0.858 (continuity corrected)
```

Supplementary Fig. 2. Begger's test for mALB

```
running c:\ado\plus\profile.do ...

. *(11 variables, 10 observations pasted into data editor)

. metan var3 var4 var5 var6 var7 var8, label(namevar=var2, yearvar=var1) random nostandard

          Study        |     WMD   [95% Conf. Interval]     % Weight
-----------------------+-----------------------------------------------------
Li et al. (2010)       |-15.965     -21.004  -10.926          7.91
Peng et al. (2013)     |-10.900     -17.083   -4.717          6.11
Xin and Ren (2013)     |-11.100     -15.107   -7.093         10.07
Wang  (2014)           |-11.210     -14.654   -7.766         11.47
Meng  (2014)           |-11.500     -17.700   -5.300          6.09
Xin  (2015)            |-14.110     -16.183  -12.037         15.31
Wang  (2016)           |-14.300     -17.785  -10.815         11.36
Wu  (2016)             |-15.840     -20.885  -10.795          7.90
Zhu  (2017)            |-15.970     -19.163  -12.777         12.14
Liu  (2017)            | -7.300     -10.677   -3.923         11.64
-----------------------+-----------------------------------------------------
D+L pooled WMD         |-12.857     -14.716  -10.999        100.00
-----------------------+-----------------------------------------------------

  Heterogeneity chi-squared =  21.02 (d.f. = 9) p = 0.013
  I-squared (variation in WMD attributable to heterogeneity) =  57.2%
  Estimate of between-study variance Tau-squared =  4.7552

  Test of WMD=0 : z=  13.56 p = 0.000
```

Supplementary Fig. 3. Meta-analysis result of mALB

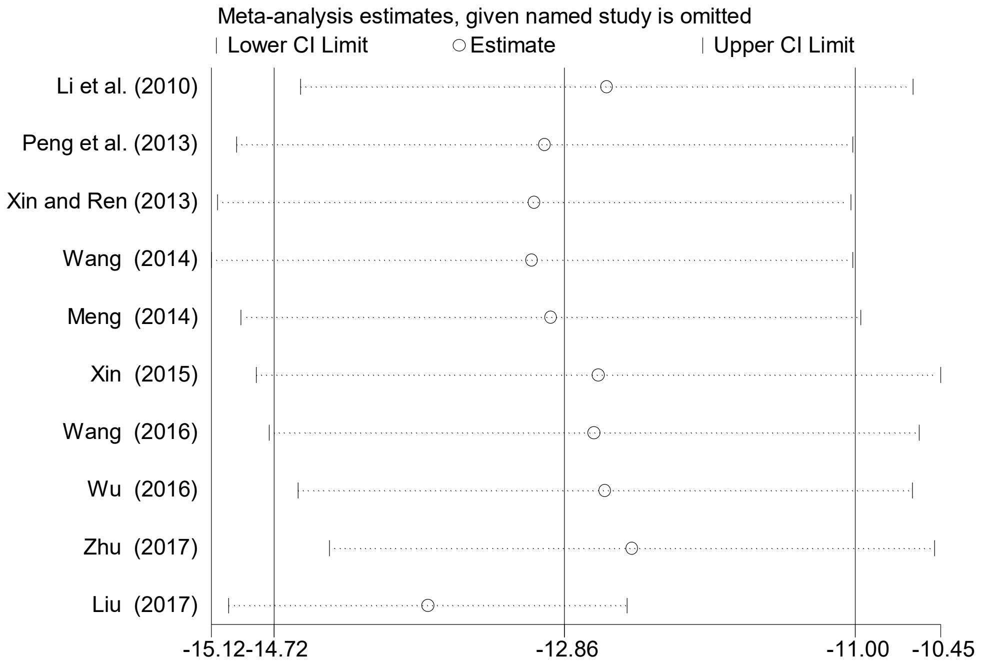

Supplementary Fig. 4. Sensitivity analysis of mALB

```
 Sub-total            |
  I-V pooled WMD      |   -7.300   -10.677    -3.923         11.27
---------------------+---------------------------------------------------
Overall               |
  I-V pooled WMD      |  -13.028   -14.162   -11.895        100.00
---------------------+---------------------------------------------------

Test(s) of heterogeneity:
               Heterogeneity  degrees of
                 statistic     freedom      P     I-squared**
With ACEI or ARB     7.13          6       0.309     15.8%
Without ACEI or ARB  0.02          1       0.893      0.0%
Not specified        0.00          0         .         .%
Overall             21.02          9       0.013     57.2%
Overall Test for heterogeneity between sub-groups:
                    13.87          2       0.001

** I-squared: the variation in WMD attributable to heterogeneity)

Some heterogeneity observed (up to 15.8%) in one or more sub-groups,
Test for heterogeneity between sub-groups may be invalid

Significance test(s) of WMD=0

With ACEI or ARB       z= 21.87      p = 0.000
Without ACEI or ARB    z=  5.01      p = 0.000
Not specified          z=  4.24      p = 0.000
Overall                z= 22.53      p = 0.000
-------------------------------------------------------------------------
```

Supplementary Fig. 5. Meta-analysis result of subgroup analysis of mALB

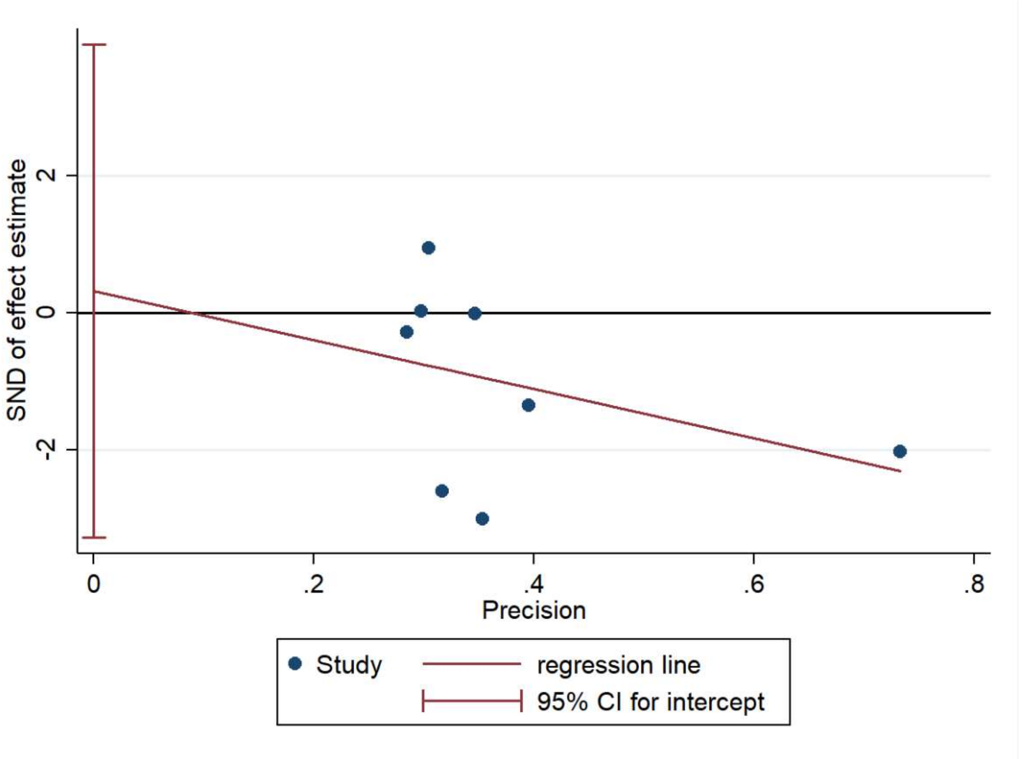

Supplementary Fig. 6. Egger's test for SBP

```
Regress standard normal deviate of intervention
effect estimate against its standard error

Number of studies =  8                              Root MSE      =   1.418

------------------------------------------------------------------------------
     Std_Eff |      Coef.   Std. Err.      t    P>|t|     [95% Conf. Interval]
-------------+----------------------------------------------------------------
       slope |  -3.578692   3.637042    -0.98   0.363    -12.47821    5.320829
        bias |   .3184964   1.465423     0.22   0.835    -3.267265    3.904258
------------------------------------------------------------------------------

Test of H0: no small-study effects          P = 0.835

. metabias _ES _seES, begg

Note: data input format theta se_theta assumed.

Begg's test for small-study effects:
Rank correlation between standardized intervention effect and its standard error

  adj. Kendall's Score (P-Q) =       6
          Std. Dev. of Score =    8.08
           Number of Studies =       8
                          z  =    0.74
                   Pr > |z|  =   0.458
                          z  =    0.62 (continuity corrected)
                   Pr > |z|  =   0.536 (continuity corrected)

.
```

Supplementary Fig. 7. Begger's test for SBP

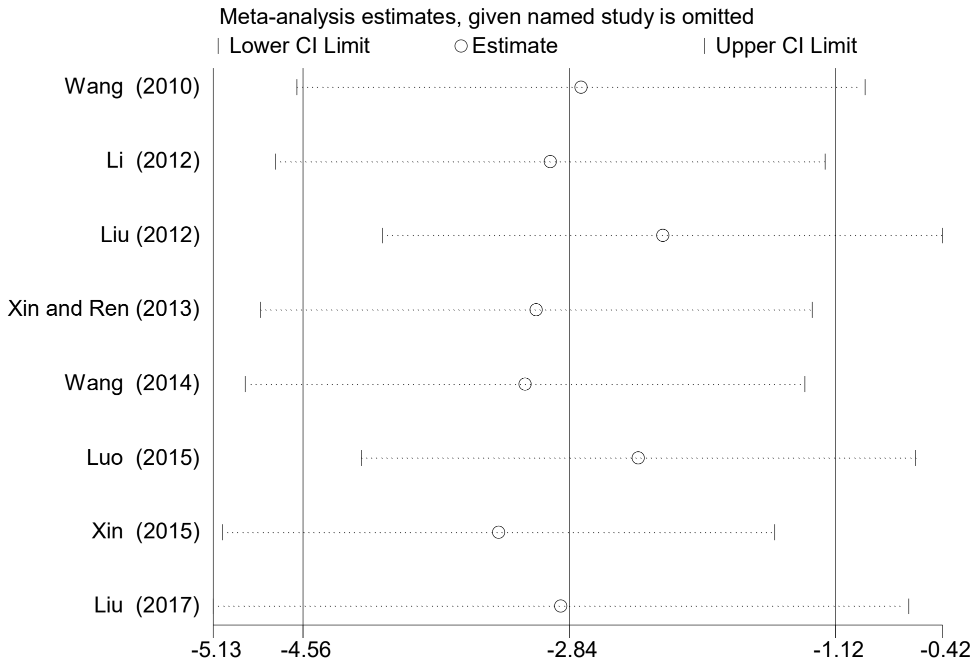

Supplementary Fig 8. Sensitivity analysis of SBP

```
. *(11 variables, 8 observations pasted into data editor)

. metan var3 var4 var5 var6 var7  var8, label(namevar=var2, yearvar=var1) fixed nostandard

           Study     |     WMD   [95% Conf. Interval]     % Weight
---------------------+---------------------------------------------------
Wang  (2010)         | -3.400      -8.364     1.564         12.00
Li  (2012)           | -1.000      -7.892     5.892          6.23
Liu (2012)           | -8.500     -14.046    -2.954          9.62
Xin and Ren (2013)   |  0.070      -6.526     6.666          6.80
Wang  (2014)         | -0.030      -5.694     5.634          9.22
Luo  (2015)          | -8.200     -14.398    -2.002          7.70
Xin  (2015)          |  3.100      -3.339     9.539          7.13
Liu  (2017)          | -2.760      -5.436    -0.084         41.30
---------------------+---------------------------------------------------
I-V pooled WMD       | -2.836      -4.556    -1.116        100.00
---------------------+---------------------------------------------------

  Heterogeneity chi-squared =  12.16 (d.f. = 7) p = 0.095
  I-squared (variation in WMD attributable to heterogeneity) =  42.4%

  Test of WMD=0 : z=   3.23 p = 0.001
```

Supplementary Fig. 9. Meta-analysis result of SBP

```
Wang  (2014)         |   -0.030    -5.694     5.634          9.22
Xin  (2015)          |    3.100    -3.339     9.539          7.13
 Sub-total           |
  I-V pooled WMD     |    0.548    -2.625     3.721         29.38
---------------------+----------------------------------------------------
Overall              |
  I-V pooled WMD     |   -2.836    -4.556    -1.116        100.00
---------------------+----------------------------------------------------

Test(s) of heterogeneity:
               Heterogeneity  degrees of
                 statistic     freedom      P    I-squared**
less than or equal t  5.12          3      0.163     41.4%
more than 4 weeks    0.86          3      0.836      0.0%
Overall             12.16          7      0.095     42.4%
Overall Test for heterogeneity between sub-groups:
                     6.19          1      0.013

** I-squared: the variation in WMD attributable to heterogeneity)

Some heterogeneity observed (up to 41.4%) in one or more sub-groups,
Test for heterogeneity between sub-groups may be invalid

Significance test(s) of WMD=0

less than or equal t  z=  4.06     p = 0.000
more than 4 weeks     z=  0.34     p = 0.735
Overall               z=  3.23     p = 0.001
--------------------------------------------------------------------------
```

Supplementary Fig. 10. *P* value of subgroup analysis of SBP

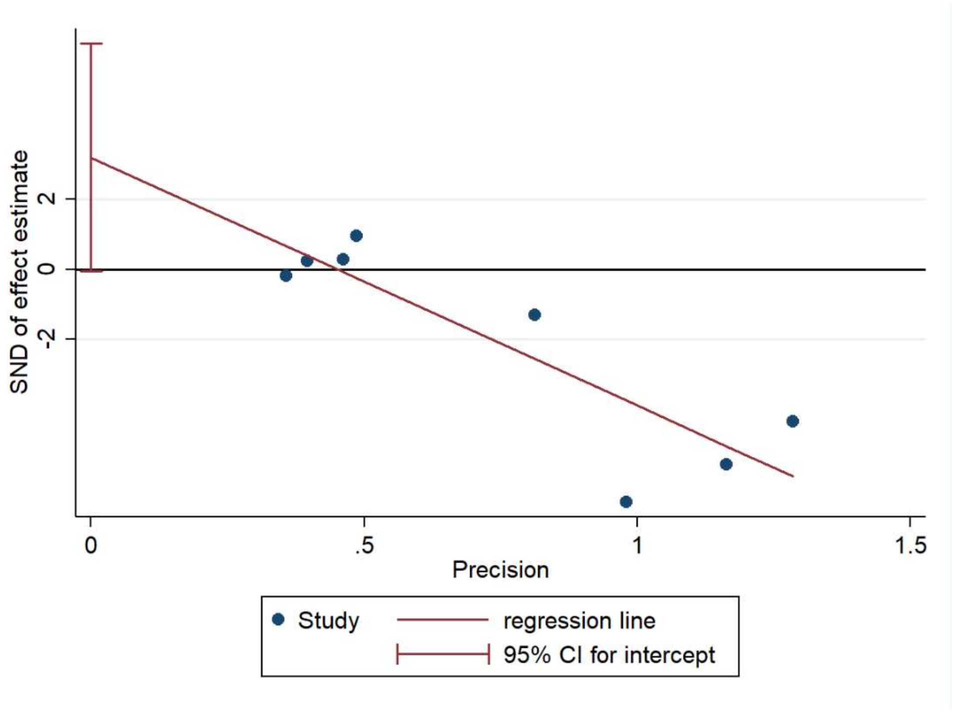

Supplementary Fig. 11. Egger's test for DBP

Std_Eff	Coef.	Std. Err.	t	P>|t|	[95% Conf.	Interval]
slope	-7.10782	1.628626	-4.36	0.005	-11.09292	-3.122716
bias	3.200196	1.333061	2.40	0.053	-.0616877	6.46208

```
Test of H0: no small-study effects          P = 0.053

. graph export "C:\Users\win10\Desktop\全文\DBP\egger-DBP.tif", as(tif) replace
(note: file C:\Users\win10\Desktop\全文\DBP\egger-DBP.tif not found)
(file C:\Users\win10\Desktop\全文\DBP\egger-DBP.tif written in TIFF format)

. metabias _ES _seES, begg

Note: data input format theta se_theta assumed.

Begg's test for small-study effects:
Rank correlation between standardized intervention effect and its standard error

  adj. Kendall's Score (P-Q) =       6
          Std. Dev. of Score =    8.08
           Number of Studies =       8
                          z  =    0.74
                    Pr > |z| =   0.458
                          z  =    0.62 (continuity corrected)
                    Pr > |z| =   0.536 (continuity corrected)

.
```

Supplementary Fig. 12. Begger's test for DBP

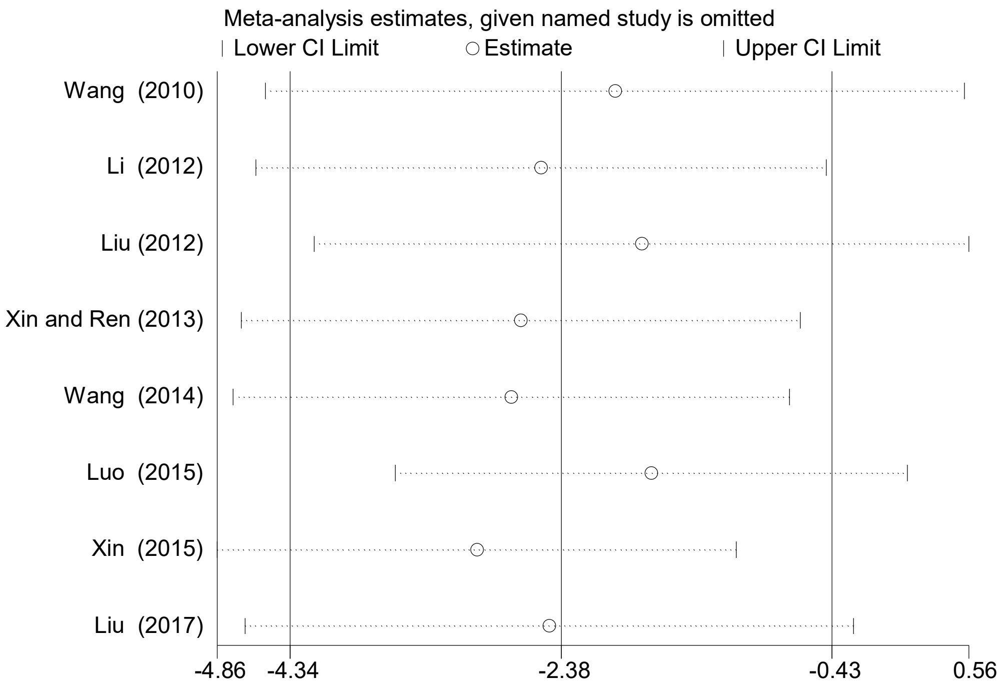

Supplementary Fig. 13. Sensitivity analysis of DBP

```
Test of H0: no small-study effects          P = 0.053

. metan var3 var4 var5 var6 var7 var8, label(namevar=var2, yearvar=var1) random nostandard

           Study     |     WMD   [95% Conf. Interval]     % Weight
---------------------+---------------------------------------------------
Wang  (2010)         | -3.400      -4.925    -1.875         16.82
Li  (2012)           | -0.500      -5.977     4.977          7.59
Liu  (2012)          | -4.800      -6.485    -3.115         16.45
Xin and Ren  (2013)  |  0.590      -4.363     5.543          8.51
Wang  (2014)         |  0.590      -3.651     4.831          9.96
Luo  (2015)          | -6.800      -8.801    -4.799         15.66
Xin  (2015)          |  1.960      -2.073     5.993         10.43
Liu  (2017)          | -1.600      -4.012     0.812         14.58
---------------------+---------------------------------------------------
D+L pooled WMD       | -2.384      -4.341    -0.427        100.00
---------------------+---------------------------------------------------

  Heterogeneity chi-squared =  29.50 (d.f. = 7) p = 0.000
  I-squared (variation in WMD attributable to heterogeneity) =  76.3%
  Estimate of between-study variance Tau-squared =  5.3213

  Test of WMD=0 : z=   2.39 p = 0.017

. graph export "C:\Users\win10\Desktop\全文\DBP\DBP-总分析.tif", as(tif) replace
(note: file C:\Users\win10\Desktop\全文\DBP\DBP-总分析.tif not found)
(file C:\Users\win10\Desktop\全文\DBP\DBP-总分析.tif written in TIFF format)

.
```

Supplementary Fig. 14. Meta-analysis result of DBP

```
---------------------+--------------------------------------------------
Overall              |
  D+L pooled WMD     |   -2.384    -4.341    -0.427        100.00
---------------------+--------------------------------------------------

Test(s) of heterogeneity:
               Heterogeneity  degrees of
                 statistic     freedom      P    I-squared**   Tau-squared

less than or equal t 12.48         3      0.006     76.0%       2.8333
more than 4 weeks     0.55         3      0.908      0.0%       0.0000
Overall              29.50         7      0.000     76.3%       5.3213
** I-squared: the variation in WMD attributable to heterogeneity)

Note: between group heterogeneity not calculated;
only valid with inverse variance method

Significance test(s) of WMD=0

less than or equal t  z=  4.33     p = 0.000
more than 4 weeks     z=  0.72     p = 0.471
Overall               z=  2.39     p = 0.017
------------------------------------------------------------------------

. graph export "C:\Users\win10\Desktop\全文\DBP\DBP-亚组分析.tif", as(tif) replace
(note: file C:\Users\win10\Desktop\全文\DBP\DBP-亚组分析.tif not found)
(file C:\Users\win10\Desktop\全文\DBP\DBP-亚组分析.tif written in TIFF format)

.
```

Supplementary Fig. 15. Meta-analysis result of subgroup analysis of DBP

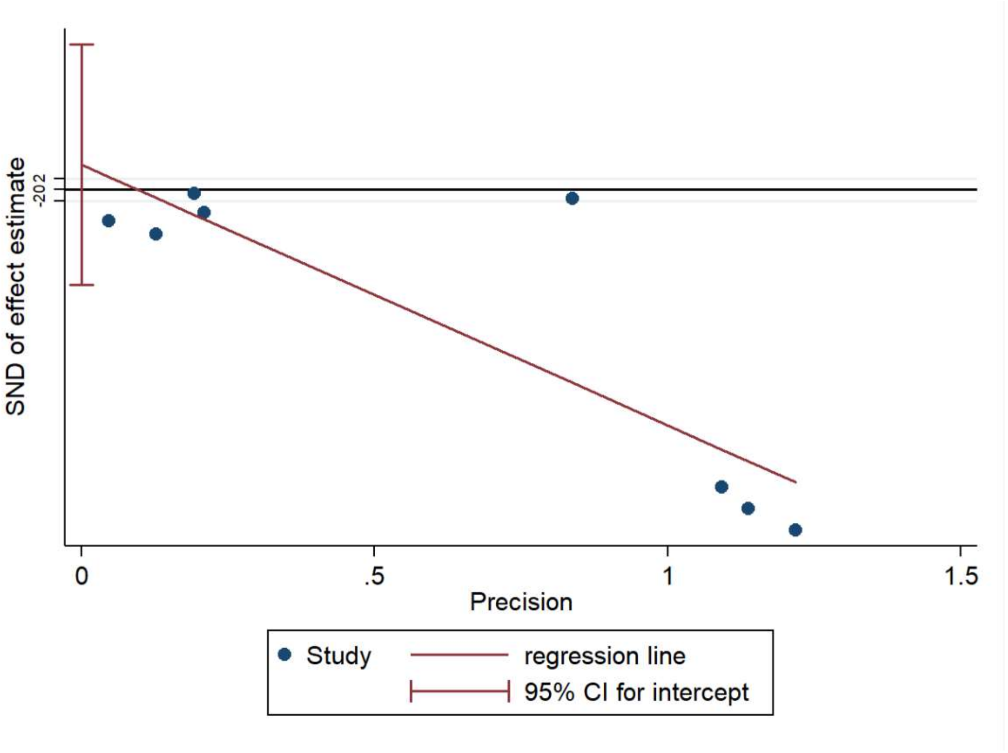

Supplementary Fig. 16. Egger's test for Scr

```
      Std_Eff |      Coef.   Std. Err.       t    P>|t|     [95% Conf. Interval]
--------------+----------------------------------------------------------------
        slope |  -48.20459      11.728    -4.11   0.006    -76.90197   -19.50721
         bias |   4.664118    9.055163     0.52   0.625    -17.49307     26.8213
-------------------------------------------------------------------------------

Test of H0: no small-study effects          P = 0.625

. graph export "C:\Users\win10\Desktop\全文\Scr\SCr-EGGER.tif", as(tif) replace
(note: file C:\Users\win10\Desktop\全文\Scr\SCr-EGGER.tif not found)
(file C:\Users\win10\Desktop\全文\Scr\SCr-EGGER.tif written in TIFF format)

. metabias _ES _seES, begg

Note: data input format theta se_theta assumed.

Begg's test for small-study effects:
Rank correlation between standardized intervention effect and its standard error

  adj. Kendall's Score (P-Q) =      10
          Std. Dev. of Score =    8.08
           Number of Studies =       8
                          z  =    1.24
                  Pr > |z|   =   0.216
                          z  =    1.11 (continuity corrected)
                  Pr > |z|   =   0.266 (continuity corrected)

.
```

Supplementary Fig. 17. Begger's test for Scr

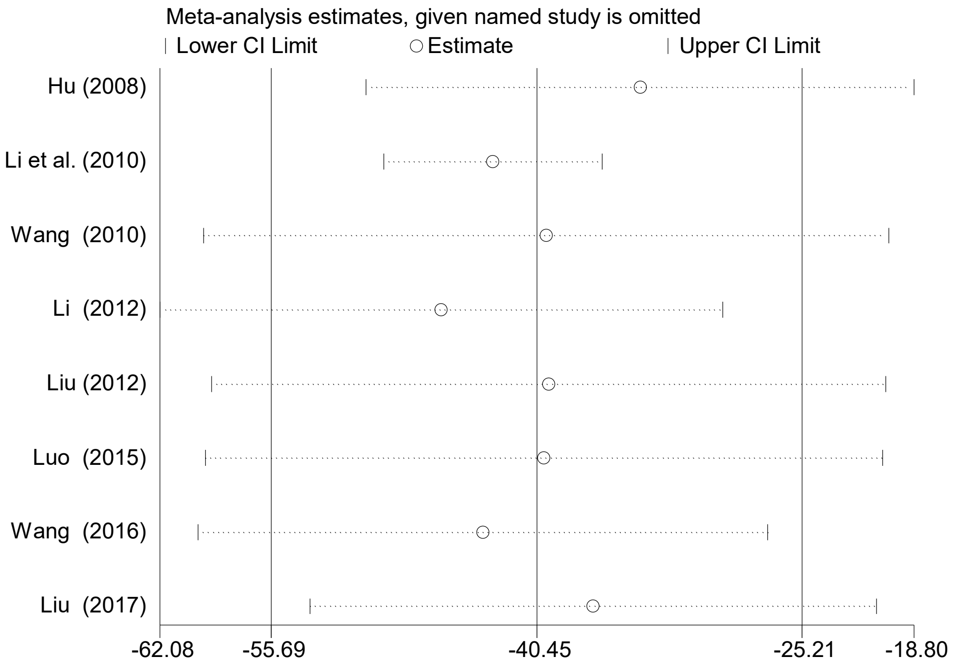

Supplementary Fig. 18. Sensitivity analysis of Scr

Std_Eff	Coef.	Std. Err.	t	P>|t|	[95% Conf.	Interval]
slope	-48.20459	11.728	-4.11	0.006	-76.90197	-19.50721
bias	4.664118	9.055163	0.52	0.625	-17.49307	26.8213

Test of H0: no small-study effects P = **0.625**

. metan var3 var4 var5 var6 var7 var8, label(namevar=var2, yearvar=var1) random nostandard

Study	WMD	[95% Conf.	Interval]	% Weight
Hu (2008)	-122.800	-164.927	-80.673	6.72
Li et al. (2010)	-1.840	-4.182	0.502	13.75
Wang (2010)	-51.600	-53.209	-49.991	13.78
Li (2012)	-3.200	-13.362	6.962	13.00
Liu (2012)	-51.900	-53.624	-50.176	13.77
Luo (2015)	-50.300	-52.094	-48.506	13.77
Wang (2016)	-19.900	-29.250	-10.550	13.12
Liu (2017)	-64.000	-79.380	-48.620	12.10
D+L pooled WMD	-40.452	-55.691	-25.212	100.00

Heterogeneity chi-squared = **1564.91** (d.f. = **7**) p = **0.000**
I-squared (variation in WMD attributable to heterogeneity) = **99.6%**
Estimate of between-study variance Tau-squared = **438.1999**

Test of WMD=0 : z= **5.20** p = **0.000**

Supplementary Fig. 19. Meta-analysis result of Scr
